# What Do Travelers Know about Traveler’s Diarrhea? Impact of a Pre-Travel Consultation in the Lisbon Area, Portugal

**DOI:** 10.3390/tropicalmed9100232

**Published:** 2024-10-08

**Authors:** Joana Estrada, Cláudia Conceição, Gonçalo Figueiredo Augusto, Rosa Teodósio

**Affiliations:** 1 NOVA National School of Public Health, Universidade NOVA de Lisboa, UNL, 1600-560 Lisbon, Portugal; 2 Public Health Unit, Local Health Unit Algarve, Rua de Antero Nobre, 8700-240 Olhão, Portugal; 3 Instituto de Higiene e Medicina Tropical, IHMT, Universidade NOVA de Lisboa, UNL, Rua da Junqueira 100, 1349-008 Lisboa, Portugal; claudiaconceicao@ihmt.unl.pt (C.C.); rosateo@ihmt.unl.pt (R.T.); 4 Global Health and Tropical Medicine, GHTM, Associate Laboratory in Translation and Innovation Towards Global Health, LA-REAL, Instituto de Higiene e Medicina Tropical, IHMT, Universidade NOVA de Lisboa, UNL, Rua da Junqueira 100, 1349-008 Lisboa, Portugal; 5 NOVA National School of Public Health, Public Health Research Centre, Comprehensive Health Research Center, CHRC, Universidade NOVA de Lisboa, UNL, 1600-560 Lisbon, Portugal; figueiredo.augusto@ensp.unl.pt

**Keywords:** traveler’s diarrhea, travel medicine, pre-travel consultations, knowledge

## Abstract

Traveler’s diarrhea (TD) is one of the most common travel-related health problems, largely interfering with planned activities and potentially contributing to antimicrobial resistance. This study aimed to characterize the knowledge about TD among pre-travel consultation users of one Portuguese travel clinic and determine the impact of the consultation on knowledge levels. Using a quasi-experimental, separate-sample pretest–posttest design, participants were randomly assigned to two groups: control/pre-consultation group (CG) or experimental/post-consultation group (EG). An anonymous self-administered questionnaire was used. A total of 470 participants were analyzed (227 CG; 243 EG). The EG/post-consultation group showed significant improvement in knowledge, with correct answers increasing from 63% to 75% (*p* < 0.001). However, knowledge gaps persisted: over 50% were unaware of TD’s self-limited nature, 30% did not recognize loperamide as a symptom reliever, and 36% believed all travelers should take antibiotics to prevent TD. The educational level and previous travel outside Europe influenced baseline knowledge; previous travel medicine consultations and information on TD improved knowledge in both groups and made it easier to acquire knowledge on the subject. Thus, a pre-travel consultation effectively increased travelers’ TD knowledge. However, post-consultation knowledge levels remained suboptimal, indicating the need for targeted interventions to increase travelers’ literacy and optimize pre-travel consultations.

## 1. Introduction

In recent decades, international travel has significantly increased. The growth in the number of international travelers because of tourism, professional travel, and migratory flows contributes to the rise of travel-related illnesses [1,2,3]. There were 1.3 billion international tourists worldwide in 2023 (a 34% increase compared to 2022). International arrivals reached 97% of the 2019 levels in the first quarter of 2024, and they are expected to reach pre-pandemic levels this year [4].

Traveler’s diarrhea (TD) has been defined in most studies as the occurrence of three or more loose stools per day, often with symptoms like fever, pain, or nausea during or shortly after travel. Bacteria cause 80–90% of cases, viruses 5–15%, and protozoa around 10% [5,6]. TD affects 30–70% of travelers over two weeks, especially in Asia, Africa, and Central/South America, due to poor sanitation and food contamination [5,7,8,9,10]. Higher TD risks are linked to shorter stays, younger age, and certain medical conditions [11,12,13]. TD is usually self-limiting, lasting 3–5 days, but it can disrupt plans and lead to post-infection issues like irritable bowel syndrome [5,6]. Treatment includes hydration, antidiarrheal medications, and antibiotics, though antibiotic use raises concerns about resistance and other side effects, such as an infection from *Clostridium difficile* [5,6,9,14].

Preventing TD involves strict hygiene, such as avoiding unbottled drinks and raw foods. Prophylactic antibiotics are not recommended; education on hygiene is crucial [5,14,15,16]. Despite accessible health advice, many travelers to tropical regions remain unaware of the risks [17,18,19,20,21].

The WHO advises a pre-travel medical consultation 4–8 weeks before departure, focusing on vaccines, medications, and risk awareness [22]. Studies show that these consultations improve knowledge, though myths persist and understanding varies by demographics [23,24,25].

In practice, loperamide and antibiotics are often prescribed for TD. The overuse of antibiotics can contribute to antimicrobial resistance, which is a concern given that 31.3% of travelers with TD in a Portuguese study used antibiotics [14,26,27,28,29].

In Portugal, there are currently 45 International Vaccination Centers (yellow fever vaccination centers) across the country, integrated into public primary health care centers, public hospitals, and clinics; these centers provide immunization and most of them also provide pre-travel consultations [30]. An undetermined number of travel medicine consultations are given in private medical clinics, both in person and online. Travel advice and the prescription of medicines or vaccines are provided only by doctors. Established in 1902, the Institute of Hygiene and Tropical Medicine (IHMT) is one of the oldest research and teaching institutions in Portugal and the single biggest provider of consultations in travel medicine in the country [2], with approximately 920 consultations per month before the pandemic and 600 per month in 2024.

This study aims to (i) characterize the knowledge about traveler’s diarrhea among users of the pre-travel consultation at the IHMT, namely its epidemiology, etiology, manifestations, treatment, and prevention; (ii) determine the impact of the advice given during the consultation on the level of travelers’ knowledge; and (iii) determine the influence of socio-demographic factors and travel variables on the level of knowledge about TD.

## 2. Material and Methods

The quasi-experimental model “separate sample pretest-posttest design” was selected [31], comparing the two samples of travelers, with the intervention being the travel medicine consultation. An anonymous self-completed questionnaire was used to obtain data and assess knowledge regarding traveler’s diarrhea. The control group answered the questionnaire before consultation; the experimental group answered it after the consultation.

### 2.1. Population and Sample

Only the travelers advised at the IHMT travel clinic, aged 18 years or older, without any cognitive, communication, or mental disorders, with oral and written comprehension of the Portuguese language, consenting to participate in the study, and signing the informed consent form, were included in the study.

Two samples were obtained (control and experimental groups) by alternately using the consultation days to obtain each of the groups. On each day, all scheduled travelers who met the inclusion criteria were invited to participate in the study. The questionnaire was applied until the desired number of participants was obtained.

According to the existing literature on the impact of carrying out travel consultations to acquire knowledge [24,27,29], the minimal effect expected in the average change in travelers’ knowledge after the consultation across all parameters is 5%, requiring a total sample of approximately 250 participants to detect this effect. Since the minimum effect estimate is not based on studies on traveler’s diarrhea in particular, and also considering the possibility of questionnaires with missing values that would have to be excluded from the analysis, it was decided to invite a total of 500 travelers who met the inclusion criteria, half for each group, to participate in the study.

### 2.2. Data Collection

The participants in the study were recruited from mid-January 2023 until the desired sample was obtained at the end of February 2023. The questionnaire was administered in the travel clinic waiting room before the consultation began or after it ended. Travelers in the control group had to complete the questionnaire before starting the consultation and were not allowed to complete it after having their consultation. Travelers in the experimental group were invited to participate in the study after completing the consultation, when they received the questionnaire, and were not allowed to complete it later outside the IHMT.

During the study, six doctors advised the travelers, all of them specialists in infectious or tropical diseases and trained in travel medicine. The doctors were not told whether travelers completed the questionnaire before or after the appointment.

### 2.3. Data Collection Instrument

The questionnaire requested information on socio-demographic characteristics (gender, age group, and educational level), previous travel experiences (previous travel outside Europe, destinations of those travels, previous travel medicine consultations), data on the travel that motivated the consultation (destinations, motive, length, motive of the booking of the consultation, previous information about TD), and 21 sentences about TD’s etiology, symptoms, transmission, prevention, and treatment with the answer options of “true”, “false”, or “do not know/do not remember”. These sentences were designed to evaluate travelers’ knowledge about TD based on the Centers for Disease Control and Prevention (CDC) recommendations and the relevant literature on traveler’s diarrhea [7].

To ensure content validity, an expert panel of three specialists in travel medicine and two specialists in public health reviewed the questions to ensure they adequately covered the epidemiology, symptoms, prevention, and treatment of TD. The internal consistency of the 21 knowledge questions was evaluated using Cronbach’s alpha, resulting in a value of 0.83, indicating good internal reliability. The questionnaire was pretested on a convenience sample of 30 travelers to assess clarity, comprehension, and applicability. Based on feedback, minor adjustments were made before full-scale data collection.

### 2.4. Data Processing and Analysis

The data were analyzed with the Statistical Package for the Social Sciences (version 25) for Windows from IBM SPSS Statistics. A statistical analysis was performed to compare the two groups using a Chi-squared test and a Chi-squared test with a Rao–Scott adjustment for questions in which participants could select more than one option. The statistical significance was set at *p* < 0.05.

Knowledge about TD was expressed as a percentage of correct answers in the questionnaire. The unanswered questions and questions answered with “I do not know” were considered to correspond to “non-knowledge” and therefore categorized as incorrect for statistical purposes.

The travel destinations were grouped according to TD risk [6] into the following categories: high-risk areas—Africa, South and Central America, Asia (except Japan and South Korea), and Oceania (except Australia and New Zealand); intermediate-risk areas—southern Europe, Israel, South Africa, the Caribbean, and the Pacific islands; low-risk areas—North America, Australia, New Zealand, Japan, South Korea, and Western Europe.

This study was conducted following the STROBE (Strengthening the Reporting of Observational Studies in Epidemiology) guidelines to ensure the comprehensive reporting of observational research. We adhered to the checklist to improve the transparency and completeness of our methods, data collection, and analysis. The limitations related to the adherence to STROBE include the fact that this is a single-site study and that there could be potential variability in the information provided by different doctors. Additionally, the study does not assess whether increased knowledge leads to changes in behavior or the incidence of traveler’s diarrhea. These limitations are further discussed in the Limitations section.

### 2.5. Ethical Considerations

This research project was submitted for consideration to the Ethical Review Board IHMT-ITQB-IGC, and a unanimous favorable opinion was issued on 18 January 2023 (ref. nº2.23).

## 3. Results

Of the 510 travelers approached to participate in the study, 13 refused to participate (2.5% of non-respondents), resulting in a total of 497 participants (243 in the pre-consultation group/control group and 254 in the post-consultation group/experimental group). Subsequently, 27 questionnaires were eliminated from the analysis because they had more than three missing answers (more than 10% missing values each), resulting in a total of 470 questionnaires being included in the analysis (227 in the control group and 243 in the experimental group).

### 3.1. Travelers’ Characteristics: Homogeneity between the Groups Studied

Table 1 shows the socio-demographic characteristics and travel experiences of the studied groups. The distribution by gender and age was similar in both groups. More than three-quarters of the participants had an educational level of a bachelor’s degree or higher (master’s/doctorate) and had previously traveled to countries outside Europe; of these, just over half had a prior travel medicine consultation.

Table 2 shows the travel characteristics of the studied groups. The most common reason for the trip that motivated the consultation was “Tourism/Leisure”, followed by “Work”, and most would travel for less than one month (more than 80%). The most common destinations were African countries followed by Asia (except South Korea and Japan) and South and Central America.

Most travelers indicated that the reason for scheduling the consultation was “Obtaining advice on prevention and treatment of illnesses during the trip” (66.4%) and to “Administer mandatory vaccines to travel to the destination” (62.8%). Around half the travelers (45.8% control group vs. 61.3% experimental group) mentioned having previously obtained information on TD, referring to “Family/Friends”, “Doctor”, and “Internet” as the main sources of information (Table 3).

No significant differences were found between the control and experimental groups regarding the characteristics of the travelers and the trips, except for obtaining information about TD on other occasions before the consultation (Table 3).

### 3.2. Knowledge about Traveler’s Diarrhea

Considering the set of questions about knowledge, on average the control group answered 63% of the questions correctly, with a significant increase in the experimental group, which answered 75% of the questions correctly (*p* < 0.001).

For most questions, knowledge increased significantly in the experimental group (Table 4 and Table 5); however, even after the consultation, less than half of the participants recognized that it is possible to have TD in any country, that this clinical condition is usually self-limited, and that in otherwise healthy adults, traveler’s diarrhea is rarely serious or life-threatening.

Regarding the food and water consumption recommendations for the prevention of TD, knowledge after the consultation reached levels above 80% on all questions (Table 4). After the consultation, only 75.3% of travelers recognized hand washing as a preventive measure for TD and the difference between the groups was not significant (Table 4).

After consultation, only 34.2% of travelers knew the usual duration of TD episodes and 43.6% knew that TD is rarely serious in healthy travelers (Table 5).

Regarding the treatment of TD, almost all travelers (92.5% in the control group, 95.9% in the experimental group) identified bloody diarrhea as a reason to see a doctor while traveling, with no significant difference between the groups. In the remaining questions relating to treatment, although knowledge had increased significantly for all of them, after the consultation, 30.5% of travelers did not recognize loperamide as an option to reduce diarrheal bowel movements; 36.2% believed that all travelers should take antibiotics to prevent TD (Table 4); 39.9% did not recognize that taking appropriate antibiotics will probably shorten the duration of diarrhea; and 27.6% incorrectly answered that any antibiotics taken on the trip can be used to treat TD (Table 5).

### 3.3. Knowledge about Traveler’s Diarrhea: Stratification by Socio-Demographic and Travel Variables

No significant differences were found regarding the percentage of correct answers between the control group and the experimental group when stratified by age, motive for traveling, and trip duration (*p* > 0.05) (Table 6).

In the control group (pre-consultation group), female participants had a significantly higher number of correct answers (69.0% vs. 57.2% in males), as did participants with higher levels of education (bachelor’s degree, master’s/doctorate) and participants who had previously traveled outside Europe (64.4% vs. 52.2%). However, these differences were no longer observed in the results after the consultation (Table 6).

In both groups, the stratified results by previous travel medicine consultation, previous information about TD, and the source of this information revealed significant differences within these subgroups, with participants who had previous travel medicine consultations and had previous information about TD—particularly those whose source of this information was a doctor—answering a greater number of questions correctly (Table 6).

## 4. Discussion

These results are consistent with those of previous studies [32], showing that pre-travel consultations are an effective strategy to increase travelers’ levels of knowledge and potentially reduce the incidence of TD and its consequences [33].

The selected study design minimized some of the threats to validity. Since each participant answered the questionnaire only once, testing effects were avoided, and the possibility of refusal decreased. The possibility of travelers being more attentive to the information given on this topic or questioning the doctor about it—not reflecting the acquisition of knowledge that would naturally occur—was also avoided.

Since the effect of the consultation is measured immediately after it is carried out, follow-up losses are avoided, as is the possible effect of other sources of knowledge on TD that could alter the outcome. However, this does not allow conclusions to be drawn about the durability of the acquisition of knowledge regarding TD. Consultations are often conducted several weeks before a trip, and it is important to understand whether the impact of the consultation persists.

Before the consultation, even among travelers with previous travel experience in risk areas and travelers who had previous travel medicine consultations, the level of knowledge was insufficient to allow for the adequate prevention and self-treatment of traveler’s diarrhea.

Regardless of the WHO recommendations to attend a pre-travel consultation before traveling to tropical regions, only about half of the participants who had previously traveled outside Europe and North America carried out a travel consultation prior to these trips. Subsequent studies are needed to assess travelers’ motives for not attending travel clinics.

Despite the significant increase in knowledge globally, gaps in knowledge about TD’s prevention and the appropriate treatment while traveling remain.

Knowledge about TD prevention after the consultation reached levels above 80% for all questions, specifically referring to recommendations on consumption of food, water, and other drinks. However, regarding hand washing as a preventative measure, only 75.3% of travelers recognized this measure as effective, and the increase in knowledge levels in the post-consultation group was not significant, suggesting that this information should be reinforced during the consultation.

Regarding TD’s treatment, although knowledge increased significantly, approximately one-third of the study participants believed that all travelers should take antibiotics to prevent TD, did not recognize that treatment with adequate antibiotics would likely shorten the duration of diarrhea, and incorrectly answered that any antibiotics brought on the trip could be used to treat TD. Collectively, the lack of knowledge on these issues may result in the misuse of antibiotics and contribute to the emergence and dissemination of antimicrobial resistance.

Another study found that the immediate recall of health issues discussed during a pre-travel consultation varies, which is consistent with the observed gaps in knowledge retention about TD prevention measures, such as hand washing, highlighting the need for reinforced messaging [34].

The results obtained after stratifying correct answers by socio-demographic characteristics and travel data suggest that, although the level of education and previous travel outside Europe may influence travelers’ baseline knowledge, they do not appear to influence the acquisition of knowledge during the consultation. The attendance at prior travel consultations and obtaining information about TD prior to the consultation, particularly when the source of this information is a doctor, appears to positively influence not only baseline knowledge but also the acquisition of knowledge during the consultation. Another study supports the current findings by showing that the acquisition of knowledge during pre-travel consultations is influenced by previous exposure to information about TD, although baseline knowledge does not entirely determine the extent of knowledge gained [23].

The groups compared in this study were homogeneous in all the socio-demographic characteristics and travel data analyzed, except for the proportion of participants who obtained information on TD before the consultation, which was higher in the post-consultation/experimental group. Since this factor influences the baseline knowledge and appears to influence the acquisition of knowledge—perhaps because these individuals were already aware of TD risk when traveling before the consultation was conducted—this difference in proportions may have contributed to the increase in knowledge in the experimental group, which may not be exclusively due to the intervention under study (the pre-travel consultation).

These results have important implications for counseling in the context of pre-travel consultations and for the prevention of TD among international travelers. Identifying gaps in knowledge and additional information factors that influence knowledge and its acquisition allows for the design of interventions aimed at adapting advice to respond to specific needs.

### Limitations

This study presents some limitations. The fact that consultations were carried out in a single travel clinic and that the consultations at the IHMT require payment may have influenced the characteristics of the participants, particularly their socioeconomic level and reasons for travel.

Since only individuals who resort to pre-travel consultations were included, baseline knowledge may not represent the population of travelers who do not resort to these consultations before traveling. Furthermore, the use of different doctors during the consultations may have introduced variability in the information provided to the participants, which was not recorded, potentially influencing post-consultation knowledge. The results may reflect different messages/content/items addressed by different doctors. The adequate training of professionals is essential to maximize the effect of consultation in knowledge acquisition.

Additionally, this study did not assess the long-term retention of knowledge or the travelers’ adherence to advice after the consultation, which could influence the prevention of traveler’s diarrhea. Subsequent studies with a different design will be necessary to characterize the impact that pre-travel advice may have on the incidence of TD.

Some studies suggest that although a pre-travel consultation reduces the morbidity associated with various infectious diseases, it does not appear to be effective in preventing TD [35]. This may be due to several factors, such as non-compliance with recommendations and the perception of diarrhea as a benign condition for which treatment is easily available. It would be important to assess whether travelers who had a pre-travel consultation experience diarrhea with identical characteristics, such as severity compared to the cases of travelers who did not have this consultation.

## 5. Conclusions

Pre-travel consultation is effective in increasing travelers’ knowledge about traveler’s diarrhea; however, several gaps persist in this knowledge. Having some information about TD before the travel consultation makes it easier to acquire knowledge on the subject. The growing prevalence of antibiotic-resistant bacteria highlights the importance of the rational use of these drugs by travelers, which can only occur if they are equipped with the adequate knowledge. Future research should focus on the development and evaluation of effective interventions to increase travelers’ literacy on TD as well as interventions aimed at maximizing the impact of pre-travel consultations, namely by adapting the recommendations to the prior knowledge of travelers as well as their socio-demographic characteristics.

Ensuring that pre-travel advice regarding the prevention and self-treatment of TD is consistent, evidence-based, and well assimilated by travelers has the potential to reduce morbidity, costs, and colonization by bacterial agents, thereby limiting the subsequent introduction of resistant organisms into the community after returning to the country of origin.

## Figures and Tables

**Table 1:** Socio-demographic characteristics and travel experience of studied groups.

Attribute		Control Group n = 227	Experimental Group n = 243	*p*-Value *
		n (%)	n (%)	
Age (years)	18–34	72 (31.7)	72 (29.6)	0.619
	35–49	72 (31.7)	67 (27.6)	
	50–65	55 (24.2)	72 (29.6)	
	>65	19 (8.4)	19 (7.8)	
	*Missing or invalid answer*	9 (4.0)	13 (5.3)	
Gender	Female	98 (43.2)	123 (50.6)	0.152
	Male	117 (51.5)	105 (43.2)	
	Other	1 (0.4)	0 (0.0)	
	*Missing or invalid answer*	9 (4.0)	15 (6.2)	
Educational level	4th grade or lower	32 (14.1)	32 (13.2)	0.922
	5th to 9th grade	3 (1.3)	2 (0.8)	
	10th to 12th grade	8 (3.5)	10 (4.1)	
	Bachelor’s degree	103 (45.4)	106 (43.6)	
	Master’s/doctorate degree	72 (31.7)	79 (32.5)	
	Other	0 (0.0)	1 (0.4)	
	*Missing or invalid answer*	9 (4.0)	13 (5)	
Previous trips outside Europe	No	32 (14.1)	28 (11.5)	0.486
	Yes	195 (85.9)	215 (88.5)	
If “yes”, prior travel medicine consultations	No	89 (45.6)	75 (34.9)	0.083
	Does not know/recall	4 (2.1)	6 (2.8)	
	Yes	102 (52.3)	134 (62.3)	
	*Missing or invalid answer*	1 (0.5)	0 (0.0)	

* Chi-squared test.

**Table 2:** Travel characteristics of studied groups.

Attribute		Control Group n = 227	Experimental Group n = 243	*p*-Value
		n (%)	n (%)	
Motive	Tourism/leisure	111 (48.9)	133 (54.7)	0.155 *
	Work	102 (44.9)	89 (36.6)	
	Others	14 (6.2)	21 (8.6)	
Length of stay	>1 month	31 (13.7)	43 (17.7)	0.291 *
	<1 month	195 (85.9)	200 (82.3)	
	*Missing or invalid answer*	1 (0.4)	0 (0)	
Destination(s)	Africa	157 (69.2)	140 (57.6)	0.139 **
	Asia (except Japan and South Korea)	48 (21.1)	72 (29.6)	
	South and Central America	25 (11.0)	29 (11.9)	
	North America	0 (0)	1 (0.4)	
	Australia, New Zealand, Japan, or South Korea	5 (2.2)	4 (1.6)	

* Chi-squared test. ** Chi-squared test with Rao–Scott correction.

**Table 3:** Motive for booking travel medicine consultation and prior information about traveler’s diarrhea.

Attribute		Control Group n = 227	Experimental Group n = 243	*p*-Value
		n (%)	n (%)	
Motive for booking the consultation	Administer mandatory vaccines	142 (62.6)	153 (63.0)	0.050 **
	Administer non-mandatory vaccines	69 (30.4)	80 (32.9)	
	Get information about vaccines	60 (26.4)	62 (25.5)	
	Obtaining advice on prevention and treatment of illnesses during the trip	137 (60.4)	175 (72.0)	
	Others	7 (3.1)	0 (0.0)	
Prior information about traveler’s diarrhea	No	105 (46.3)	81 (33.3)	0.005 *
	Does not know/recall	18 (7.9)	12 (4.9)	
	Yes	104 (45.8)	149 (61.3)	
	*Missing or invalid answer*	0 (0.0)	1 (0.4)	
If “Yes” source of the prior information about traveler’s diarrhea	Doctor	39 (37.5)	86 (57.7)	0.296 **
	Nurse	2 (1.9)	5 (3.4)	
	Family/friends	61 (58.7)	71 (47.7)	
	School	5 (4.8)	10 (6.7)	
	Internet	30 (28.8)	46 (30.9)	
	Media	8 (7.7)	13 (8.7)	
	Others	10 (9.6)	11 (7.4)	

* Chi-squared test. ** Chi-squared test with Rao–Scott correction.

**Table 4:** Knowledge about risk factors and prevention of traveler’s diarrhea.

Question ^†^	Correct Answer	Percentage of Correct Answers		*p*-Value *
		Control Group (n = 227)	Experimental Group (n = 243)	
Traveler’s diarrhea is a rare travel-related illness	False	52.0%	72.8%	<0.001
Traveler’s diarrhea can be caused by infectious agents	True	55.5%	74.9%	<0.001
The risk of getting traveler’s diarrhea only exists in some countries	False	30.8%	42.8%	0.007
The risk of getting traveler’s diarrhea by drinking tap water in most countries in Asia, Africa and Central and South America is low	False	85.9%	95.5%	<0.001
Consuming drinks with ice increases the risk of traveler’s diarrhea	True	74.4%	94.2%	<0.001
The risk of getting traveler’s diarrhea by drinking beverages from factory-sealed containers is low	True	73.6%	90.5%	<0.001
Consuming raw vegetables increases the risk of traveler’s diarrhea	True	84.6%	98.4%	<0.001
Consuming partially uncooked/rare meat can cause traveler’s diarrhea	True	75.3%	79.8%	0.241
Washing and peeling the fruit before eating it decreases the risk of traveler’s diarrhea	True	72.7%	82.7%	0.009
Washing your hands frequently is an effective measure to reduce the risk of traveler’s diarrhea	True	69.2%	75.3%	0.137
Boiling water and other drinks before consuming them can help reduce the risk of traveler’s diarrhea	True	82.8%	87.2%	0.178
All travelers should take antibiotics to prevent traveler’s diarrhea	False	42.7%	63.8%	<0.001

^†^ Questions originally presented to participants in Portuguese. * Chi-squared test.

**Table 5:** Knowledge about clinical presentation and treatment of traveler’s diarrhea.

Question ^†^	Correct Answer	Percentage of Correct Answers		*p*-Value *
		Control Group (n = 227)	Experimental Group (n = 243)	
Traveler’s diarrhea usually lasts 3 to 5 days if untreated	True	28.2%	34.2%	0.164
In otherwise healthy adults, traveler’s diarrhea is rarely serious or life-threatening	True	35.2%	43.6%	0.063
Symptoms of traveler’s diarrhea may include abdominal pain, cramps, and nausea	True	79.3%	89.3%	0.003
Symptoms of traveler’s diarrhea may include fever	True	55.1%	69.1%	0.002
If you have traveler’s diarrhea you should drink lots of fluids	True	72.2%	83.5%	0.003
Loperamide/Imodium can be used to reduce frequency of bowel movements	True	55.5%	69.5%	0.002
If you have bloody stools, you should see a doctor	True	92.5%	95.9%	0.116
Taking an adequate antibiotic will likely shorten the duration of the traveler’s diarrhea	True	37.4%	60.1%	<0.001
Any antibiotic you carry on the trip can be used to treat traveler’s diarrhea	False	60.8%	72.4%	0.007

^†^ Questions originally presented to participants in Portuguese. * Chi-squared test.

**Table 6:** Percentage of correct answers in the control and experimental groups stratified by socio-demographic and travel variables.

Attribute	Control Group		Experimental Group	
	Correct Answer (%)	*p*-Value *	Correct Answer (%)	*p*-Value *
Age (years)		0.829		0.776
18–34	60.5		76.0	
35–49	62.3		73.1	
50–65	66.7		76.0	
>65	59.9		74.7	
Gender		<0.001		0.218
Female	69.0		76.4	
Male	57.2		73.3	
Educational level		0.014		0.320
4th grade or lower	57.1		69.0	
5th to 9th grade	45.8		71.9	
10th to 12th grade	58.2		68.9	
Bachelor’s degree	63.2		76.0	
Master’s/doctorate degree	65.7		76.7	
Previous trips outside Europe		<0.001		0.053
Yes	64.4		75.9	
No	52.2		68.5	
Prior travel medicine consultations		<0.001		0.009
Yes	67.7		78.6	
No	61.3		71.4	
Motive		0.060		0.766
Tourism/leisure	64.1		75.8	
Work	60.1		74.3	
Others	70.1		73.2	
Length of stay		0.688		0.361
>1 month	64.2		72.6	
<1 month	62.6		75.5	
Prior information about TD		<0.001		<0.001
Yes	73.5		79.5	
No	53.1		67.7	
Source of the prior information		<0.001		0.003
Doctor	77.0		79.9	
All other sources	59.7		72.4	

* Chi-squared test. TD: traveler’s diarrhea.

## Data Availability

The data presented in this study are available on request from the corresponding author due privacy reasons.
